# Prebiotic Wheat Bran Fractions Induce Specific Microbiota Changes

**DOI:** 10.3389/fmicb.2018.00031

**Published:** 2018-01-24

**Authors:** Kevin D’hoe, Lorenza Conterno, Francesca Fava, Gwen Falony, Sara Vieira-Silva, Joan Vermeiren, Kieran Tuohy, Jeroen Raes

**Affiliations:** ^1^Laboratory of Molecular Bacteriology, Department of Microbiology and Immunology, Rega Institute, KU Leuven, Leuven, Belgium; ^2^Jeroen Raes Lab, VIB KU Leuven Center for Microbiology, Leuven, Belgium; ^3^Research Group of Microbiology, Department of Bioengineering Sciences, Vrije Universiteit Brussel, Brussels, Belgium; ^4^Department of Food Quality and Nutrition, Research and Innovation Centre, Fondazione Edmund Mach, Trento, Italy; ^5^Fermentation and Distillation, Laimburg Research Centre, Bolzano, Italy; ^6^Cargill R&D Centre Europe BVBA, Vilvoorde, Belgium

**Keywords:** wheat bran, aleurone, prebiotic, *in vitro*, fermentation, microbiome

## Abstract

Wheat bran fibers are considered beneficial to human health through their impact on gut microbiota composition and activity. Here, we assessed the prebiotic potential of selected bran fractions by performing a series of fecal slurry anaerobic fermentation experiments using aleurone as well as total, ultrafine, and soluble wheat bran (swb) as carbon sources. By combining amplicon-based community profiling with a fluorescent *in situ* hybridization (FISH) approach, we found that incubation conditions favor the growth of Proteobacteria such as *Escherichia* and *Bilophila*. These effects were countered in all but one [total wheat bran (twb)] fermentation experiments. Growth of *Bifidobacterium* species was stimulated after fermentation using ultrafine, soluble, and twb, in the latter two as part of a general increase in bacterial load. Both ultrafine and swb fermentation resulted in a trade-off between *Bifidobacterium* and *Bilophila*, as previously observed in human dietary supplementation studies looking at the effect of inulin-type fructans on the human gut microbiota. Aleurone selectively stimulated growth of *Dorea* and butyrate-producing *Roseburia*. All fermentation experiments induced enhanced gas production; increased butyrate concentrations were only observed following soluble bran incubation. Our results open perspectives for the development of aleurone as a complementary prebiotic selectively targeting colon butyrate producers.

## Introduction

Although amplicon sequencing approaches are nowadays applied routinely to study the bacterial composition of the gut microbiota (Valles-Colomer et al., 2016), their application in prebiotic food ingredient research has been lagging behind (Hutkins et al., 2016). Only recently, 16S rRNA gene sequencing was applied to map the compositional changes induced by a dietary intervention using inulin-type fructans (Vandeputte et al., 2017). A community-wide analysis estimated the effect size of this prebiotic intervention in relative microbiome composition to 1.2% (Vandeputte et al., 2017) – modest, but comparable to the impact of top covariates of microbiome variation such as whole bread consumption (Falony et al., 2016). Unexpectedly, the study confirmed selective stimulation of a limited number of bacterial taxa upon inulin consumption, in line with the original definition of a prebiotic substrate (Gibson and Roberfroid, 1995). This observation has challenged the emerging scientific consensus regarding a community-level response of the gut microbiota to prebiotic interventions (Claus, 2017). It will most likely revive efforts to identify complementary food ingredients, selectively stimulating growth or activity of distinct sets of intestinal bacteria with potential beneficial properties.

A key aspect in maintaining gastrointestinal health lies in the consumption of dietary fiber. Fiber does not only accelerate intestinal transit (Burkitt et al., 1972), it also provides a variety of fermentable substrates to the intestinal microbiota (Sonnenburg and Sonnenburg, 2014). Fermentation of fiber polysaccharides increases microbiota production of short chain fatty acids (De Filippis et al., 2016), including butyrate. The latter not only represents the main energy source for colon epithelial cells (Roediger, 1982), it also affects cellular differentiation processes and has been shown to exert anti-inflammatory effects (Hamer et al., 2008; Louis et al., 2014). Moreover, the availability of readily fermentable, fiber-derived polysaccharides reduces potentially deleterious proteolytic fermentation processes (De Preter et al., 2010) and restrains microbial erosion of the mucus layer (Desai et al., 2016). By promoting growth and activity of commensal micro-organisms, dietary fiber consumption also reduces the risk of pathogenic invasion, both by enhancing colonization resistance and decreasing luminal pH (Lawley and Walker, 2013).

Wheat bran represents one of the main contributors to daily fiber intake in Western diets (Stevenson et al., 2012). Specific components of bran fiber such as arabinoxylans (Broekaert et al., 2011) have been shown to induce a prebiotic effect in the gut microbial ecosystem. Here, we contribute to the pursuit of novel potential prebiotics by assessing compositional and metabolic changes in fecal slurries upon fermentation of selected wheat bran fractions.

## Results

### Fecal Fermentations Impact Microbiota Composition

To assess the prebiotic potential of different wheat bran fractions, a series of 18 fecal fermentations experiments was set up using fecal material donated by six healthy volunteers (Supplementary Table S1). Selected bran fractions comprised total wheat bran (twb), ultrafine wheat bran (uwb), soluble wheat bran (swb), and aleurone (alr) (**Table 1**). Fermentations were carried out in triplicate; each replicate was inoculated with fecal material from a different donor. In parallel, cellulose incubations were set up with fecal material from each volunteer. Given its limited fermentability by colon bacteria (Mudgil and Barak, 2013), in these control cellulose fermentations, the effect of experimental conditions was expected to dominate over the impact of substrate fermentation. Hence, they allowed assessing the impact of the experimental set-up on the fecal microbiota composition. Overall, 24 h cellulose fermentation resulted in a decrease of genus richness when compared to donor material [paired *t*-test, effect size (ES) = -0.66, *p*-value = 8.1 × 10^-3^; Supplementary Figure S1]. Changes in taxa abundances included the increased relative abundances of *Escherichia* (paired *t*-test, ES = 0.97, FDR = 7.0 × 10^-5^), *Bilophila* (ES = 0.92, FDR = 3.3 × 10^-4^), and *Sutterella* (ES = 0.62, FDR = 2.8 × 10^-2^; Supplementary Table S2). In contrast, proportions of *Roseburia* (paired *t*-test, ES = -0.95, FDR = 1.6 × 10^-4^), *Bacteroides* (ES = -0.61, FDR = 2.8 × 10^-2^), *Faecalibacterium* (ES = -0.54, FDR = 4.9 × 10^-2^), and *Blautia* (ES = -0.47, FDR = 6.4 × 10^-2^) were reduced (Supplementary Table S2). Hence, in summary, experimental conditions were shown to result in a decrease of bacterial richness, mostly due to proportional blooming of Proteobacteria.

**Table 1:** Chemical composition of total wheat bran, ultrafine wheat bran, wheat aleurone, and soluble wheat bran (in %; mean values, rsd < 5%).

	Total wheat bran	Ultrafine wheat bran	Aleurone	Soluble wheat bran
Moisture (%)	11	7.7	6.6	2.1
Total protein (%)	17	18	17	0.5
Total fat (%)	/	/	5.5	/
Starch (%)	26	27	6.3	/
Ash (%)	5.2	5.1	7.2	0.4
Fiber	38	37	56	79
Insoluble fiber	34	33	48	/


### Donor and Substrate Diversify the Outcomes of Fecal Slurry Fermentations

**FIGURE 1:**
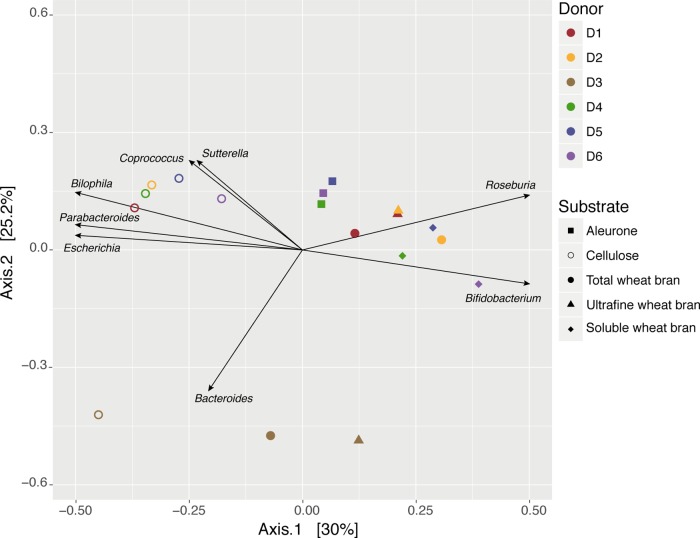
Genus-level microbiome community variation of fecal slurry incubation experiments after 24 h, represented by principal coordinates analysis (Bray–Curtis dissimilarity PCoA). Samples (*n* = 18) were colored and shaped by donor and substrate, respectively. Genera that displayed significant variation among samples (db-RDA, FDR < 0.10) were scaled according to contribution and plotted on the ordination. The percentage of variance explained by the two first PCoA dimensions are reported on the axes.

Next, we assessed donor- and substrate-specific variation through the analysis of sample microbiome dissimilarity after 24 h of fermentation. A principal component analysis (PCoA) was used to visualize the between-samples dissimilarity in terms of microbiota composition (Bray–Curtis dissimilarity), revealing separate clusters of cellulose incubations and fermentations inoculated with donor 3 (D3) fecal material (**Figure 1**). These observations were confirmed by a hierarchical clustering approach, with the cellulose and D3 subgroups branching out at higher levels. D3 sample grouping clearly originated from *Acidaminococcus* blooming. Even though only representing a minor fraction of the donor microbiota, D3 fecal material was characterized by elevated *Acidaminococcus* relative abundances [unpaired *t*-test, (ES) = 0.99, *p*-value = 5.7 × 10^-9^; overall donor variation in core taxa (present in 80% of samples, abundance > 5% in at least one sample) is summarized in Supplementary Table S3 and Figure S2]. Clustering of cellulose incubations did suggest substrate-driven microbiome differentiation of bran fraction fermentations. Overall, substrate and donor explained, respectively, between 25–48 and 6–40% of microbiome variation during incubation experiments (Supplementary Table S4).

### Bran Fraction Fermentations Do Not Differentially Alter Community Richness

**FIGURE 2:**
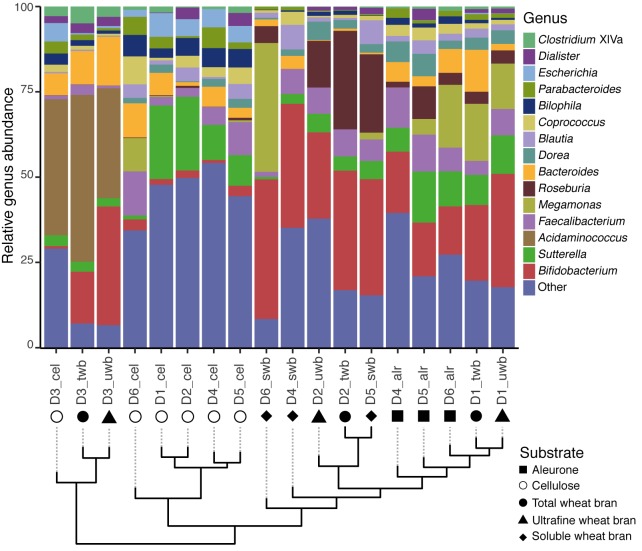
Genus level relative microbiome composition of fecal slurry incubation experiments. Samples are clustered based on Bray–Curtis dissimilarity. The top 15 classified genera are depicted, with all others pooled into ‘Other.’ Clustering analysis was performed by hierarchical clustering with mean linkage.

Fecal microbiome richness has been put forward as a read-out of colonic microbiota stability or resilience (Vieira-Silva et al., 2016), with reduced estimates thought to be indicative for ecosystem dysbiosis (Qin et al., 2012; Le Chatelier et al., 2013). However, some recent findings have associated high community richness indices to hard stools (Vandeputte et al., 2016), prolonged transit times (Roager et al., 2016), and enhanced proteolytic fermentation (Macfarlane et al., 1989; Roager et al., 2016), suggesting a less straightforward association between richness and host health than generally assumed. Given the interest in prebiotic modulation of microbiota richness (Druart et al., 2014; Bindels et al., 2015; Vandeputte et al., 2017), we assessed the impact of substrate variation on the number of observed genera following 24 h fecal slurry incubations (**Figure 2**). We did not observe any shift in richness associated with incubation of cellulose or any of the selected wheat bran fractions (Kruskal–Wallis test, *p*-value = 0.21; Supplementary Figure S3). In contrast, donor material did moderately affect the outcome of fermentation experiments (Kruskal–Wallis, *p*-value = 6.3 × 10^-2^; Supplementary Figure S4) – which could, however, be attributed to reduced richness in D3 incubations (Dunn’s test; Supplementary Table S5).

### Bran Fraction Fermentations Suggest Specific Prebiotic Effects on Gut Microbiota

Using an *in vitro* incubation approach, the prebiotic potential of a substrate can only be deduced from its ability to stimulate beneficial bacteria upon fermentation. To identify substrate-specific responsive genera, we compared taxa relative abundances after 24 h of bran fermentations with the outcome of cellulose incubations in matching donor fecal slurries (Supplementary Table S6). On genus level, wheat bran fractions were found to induce specific changes in slurry microbiota compositions. While all bran fraction fermentations resulted in increased *Bifidobacterium* relative abundances (paired *t*-test, twb, ES = 0.99, FDR = 2.6 × 10^-2^; uwb, ES = 0.91, FDR = 9.8 × 10^-2^; swb, ES = 0.90, FDR = 5.4 × 10^-2^; alr, ES = 0.77, FDR = 9.5 × 10^-2^), alr microbiome profiles were additionally characterized by higher proportions of both *Roseburia* (ES = 0.92, FDR = 5.9 × 10^-2^) and *Dorea* (ES = 0.80, FDR = 9.5 × 10^-2^). In contrast, the genera *Escherichia* (swb, ES = -0.98, FDR = 1.9 × 10^-2^; alr, ES = -1.0, FDR = 7.9 × 10^-4^), *Parabacteroides* (swb, ES = -0.99, FDR = 9.7 × 10^-3^; alr, ES = -0.88, FDR = 6.6 × 10^-2^), and *Bilophila* (swb, ES = -0.94, FDR = 3.9 × 10^-2^; alr, ES = -0.91, FDR = 5.9 × 10^-2^) were underrepresented following swb and alr fermentation. While a similar decrease in *Parabacteroides* relative abundances could be observed in twb fermentation (ES = -0.97, FDR = 3.3 × 10^-2^), uwb fermentation outcomes only mirrored reduced *Bilophila* populations (ES = -0.98, FDR = 3.8 × 10^-2^). In general, the addition of wheat bran fractions to fecal slurries resulted in increased *Bifidobacterium* relative abundances – mostly at the expense of the Proteobacteria, shown to be favored by incubation conditions. Of all substrates under investigation, alr displayed the broadest impact range on microbiota composition. Remarkably, the *Bifidobacterium*/*Bilophila* trade-off observed in uwb, swb, and alr incubations matched particularly well microbiota fluctuations following inulin consumptions observed *in vivo* (Vandeputte et al., 2017).

### FISH Taxon Enumeration Confirms Bifidogenic Effect of Bran Fractions

**FIGURE 3:**
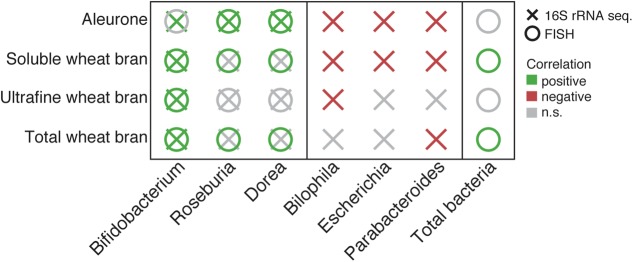
Summary of significant changes in core taxa relative abundances following bran fraction incubation experiments compared to cellulose fermentations as detected through 16S rRNA gene amplicon sequencing. Quantitative validation of results was performed using a FISH approach (18 samples; paired *t*-test, statistics are included in Supplementary Tables S6, S7).

Given the compositional nature of microbiome data, comparative analyses such as described above cannot reveal absolute directionality of observed abundance fluctuation. To bypass this problem, we performed a validation experiment using a FISH approach (**Figure 3** and Supplementary Table S7). Given pretreatment and nature of the substrates studied, overall increases in total bacterial abundances were mostly limited (Supplementary Table S1). FISH enumeration data for bifidobacteria were found to correlate surprisingly well with relative abundances obtained through amplicon sequencing (Pearson, *r* = 0.83, *p*-value = 2.4 × 10^-5^). Except for alr, absolute quantification allowed us to confirm the bifidogenic effect of twb (paired *t*-test, ES = 0.90, *p*-value = 1.9 × 10^-2^), uwb (ES = 0.97, *p*-value = 4.8 × 10^-3^), and swb (ES = 0.81, *p*-value = 4.0 × 10^-2^; Supplementary Table S7). The association observed between alr and *Roseburia*/*Dorea* relative abundances was also validated (ES = 0.87, *p*-value = 2.5 × 10^-2^) based on the abundances detected using the FISH *Eubacterium rectale*/*Clostridium coccoides* spp (EREC) probe (Duncan et al., 2007). Of note, total and soluble bran fermentations also resulted in stimulated growth of EREC taxa (twb, ES = 0.75, *p*-value = 5.9 × 10^-2^; swb, ES = 0.94, *p*-value = 1.1 × 10^-2^), which remained unnoticed using a compositional sequencing approach – most probably due to the overall increase of bacterial numbers following both twb (ES = 0.65, *p*-value = 9.8 × 10^-2^) and swb (swb, ES = 0.81, *p*-value = 4.0 × 10^-2^) incubation.

### Incubation of Soluble Wheat Bran Stimulates Butyrate Production

**FIGURE 4:**
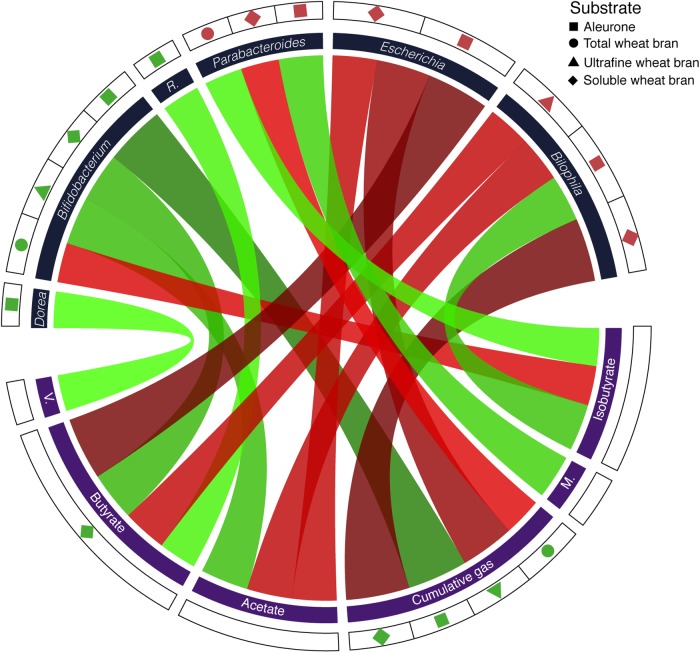
Circos plot depicting correlations between substrates, metabolites, and relative genus abundances [FDR < 10% for core taxon (*n* = 14) – substrate (*n* = 4) associations; FDR < 10% for core taxon (*n* = 4; only correlations of taxa with increased relative abundances following bran fermentations were included) – metabolite (*n* = 8) correlations; *p*-value < 5% for metabolite (*n* = 8) – substrate (*n* = 4) associations]. Green and red ribbons represent positive and negative relationships, respectively. Ribbons are sized and colored according to the strength of the association (effect size). R, *Roseburia*; M, methylbutyrate; V, valerate.

In all fermentation experiments, we quantified acetate, propionate, butyrate, lactate, valerate, isobutyrate, and methylbutyrate concentrations as well as total gas production after 24 h of substrate fermentation. Based on the metabolite profiles, dissimilarity between wheat bran and cellulose fermentations was assessed (Euclidean distance). Metabolite dissimilarity was found strongly correlated to sample microbiome differentiation (Bray–Curtis) (Mantel test, *r* = 0.24, *p*-value = 1.6 × 10^-2^). Hence, changes in taxa relative abundances were reflected in the metabolic output of fecal slurry fermentation processes (Supplementary Figure S5). Compared to cellulose, fermentations of selected wheat bran fractions gave rise to increased gas production (twb, paired *t*-test, ES = 0.99, *p*-value = 2.2 × 10^-3^; uwb, ES = 0.98, *p*-value = 3.8 × 10^-3^; swb, ES = 0.98, *p*-value = 4.1 × 10^-3^; alr, ES = 0.83, *p*-value = 3.7 × 10^-2^; Supplementary Table S8). Moreover, swb fermentations displayed elevated butyrate concentrations (ES = 0.88, *p*-value = 2.3 × 10^-2^), matching FISH findings regarding EREC cluster absolute counts (Hold et al., 2003). Interestingly, independent of donor or substrate variation, relative abundances of *Bifidobacterium*, *Bilophila*, *Escherichia*, and *Parabacteroides* could be correlated with several metabolites, including acetate, butyrate, isobutyrate, and cumulative gasses (Supplementary Table S9). In contrast, *Roseburia* could only be associated to butyrate, in line with the genus’ metabolic profile (Duncan et al., 2002). *Dorea*, on the other hand, was exclusively linked with valerate concentrations – although the taxon is known as a major gas producer (Rajilić-Stojanović and de Vos, 2014). An overview of substrate, taxon, metabolite associations observed in bran fraction incubation experiments is presented in **Figure 4**.

## Discussion

Our *in vitro* incubation analyses largely confirmed the bifidogenic effect of total, ultrafine, and swb fractions (Cloetens et al., 2010; Neyrinck et al., 2011; François et al., 2012; Maki et al., 2012). The strategies and mechanisms applied by *Bifidobacterium* spp. to degrade non-digestible carbohydrates have previously been described in detail (Rivière et al., 2014, 2016). Moreover, wheat fiber is constituted primarily of arabinoxylans (Grootaert et al., 2007; Hughes et al., 2007; Vardakou et al., 2008) with known dose-dependent bifidogenic properties (Cloetens et al., 2010; Neyrinck et al., 2011; François et al., 2012; Maki et al., 2012). Although end-products of bifidobacterial carbohydrate metabolism are mainly limited to lactate, acetate, formate, and ethanol (Rivière et al., 2016), the genus has been shown to sustain a broad range of gut microbial commensals through cross-feeding interactions (Duncan et al., 2004; Belenguer et al., 2006; Falony et al., 2006; Moens et al., 2016, 2017). The latter would expand the metabolic impact of stimulated *Bifidobacterium* growth to include the observed increase in butyrate and gas production (De Vuyst and Leroy, 2011).

Given its putative health-promoting properties (Scheppach and Weiler, 2004), the enhancement of colonic butyrate production has been a long-standing target of prebiotic research. Here, we noted increased abundance of *Roseburia* spp. following fermentation of both swb and aleurone. Butyrate-producing *Roseburia* spp. have been shown to be able to grown on inulin (Scott et al., 2014), xylans (Duncan et al., 2002; Chassard et al., 2007), and arabinoxylans (Sheridan et al., 2016), as well as on intermediates of primary polysaccharide degradation (Belenguer et al., 2006; Falony et al., 2006). While increased abundance of *Roseburia* following soluble fiber incubation was part of a more generalized stimulation of total bacterial growth, the effect induced by aleurone appeared more taxon-specific. Aleurone has previously been attributed bifidogenic properties (Brouns et al., 2012). Here, using a community-wide analytical approach, we demonstrate that it could potentially be applied for targeted stimulation of *Clostridium* cluster IVa bacteria, encompassing several colon butyrate producers.

In all but one (twb) incubation experiments, fermentation of bran fractions resulted in decreased Proteobacteria taxa when compared to cellulose. Besides the fact that these genera are known to thrive under *in vitro* conditions, their proteolytic or aminoacidolytic nature provides them with a selective advantage when incubated in the absence of readily fermentable carbohydrates. While their decrease in relative abundances could partially result from compositionality effects, it also demonstrates the potential of refined bran fractions to extend saccharolytic fermentation to more distal gut regions. Given the production of potentially deleterious components resulting from proteolytic fermentation (Hamer et al., 2011), the latter is considered a desirable property of functional food ingredients targeting the microbial gut ecosystem (Macfarlane et al., 2006). The effects of ultrafine or swb incubations on *Bilophila* relative abundances matched remarkably well previous *in vivo* findings regarding the prebiotic properties of inulin (Vandeputte et al., 2017). The most prominent intestinal *Bilophila* isolate is *Bilophila wadsworthia*, an asaccharolytic sulfate reducer that has been characterized as an opportunistic pathogen (Baron et al., 1989; Baron, 1997; da Silva et al., 2008). Our *in vitro* findings suggest that the trade-off between the *Bifidobacterium* and *Bilophila* taxa is a consequence of direct bacterial interactions (e.g., competition or metabolite production) rather than a host-mediated response to a dietary intervention or intervention-induced primary shifts in microbiota composition as suggested previously (Vandeputte et al., 2017). Overall, the results presented indicate that similar bacterial interactions make up the core of the microbiota’s colonization resistance against non-commensal intruders – a phenomenon that appears to be strengthened by the availability of fermentable substrates, as suggested by the restrained blooming of Proteobacteria in bran incubations. The fermentable fibers present in the wheat bran fractions studied provide the commensal microbiota with a competitive advantage, hampering settlement of opportunistic colonizers. In this pH-controlled setting, we observed colonization resistance to be independent of total bacterial abundance. The role of bacterial metabolites produced upon fiber fermentations in this process remains to be elucidated.

Finally, bran supplementation also altered the outcome of niche competition between saccharolytic taxa. *Parabacteroides* spp., part of a normal large-intestinal microbiota but often associated with opportunistic infections (Nakano et al., 2011), have been described to bloom on resistant starches (Martínez et al., 2010) rather than on complex non-starch polysaccharides (Sakamoto and Benno, 2006). Except for uwb, fecal fermentations of bran fractions provided a growth advantage to saccharolytic competitors such as bifidobacteria, allowing them to dominate over the *Parabacteroides* fraction.

## Materials and Methods

### Donor Fecal Material

Fecal material was collected from six healthy, male subjects aged between 30 and 47, not receiving antibiotic treatment for at least 3 months, not consuming pre- or probiotic containing supplements prior to experimentation, and without history of intestinal disorders. Fecal slurry was prepared under anaerobic conditions by homogenizing fresh human fecal material in ten times the volume of pre-reduced phosphate buffered saline (PBS; 8 g/L NaCl, 0.2 g/L KCl, 1.15 g/L Na_2_PO_4_, and 0.2 g/L KH_2_HPO_4_).

### Substrates

Total wheat bran (twb), uwb, swb, and aleurone (alr) were used. Uwb was obtained through mechanical milling of twb, resulting in particles with a size < 100 μm. Swb was obtained through enzymatic treatment of wheat bran, reducing arabinoxylan chain length into shorter oligosaccharides (arabinoxylan-oligosaccharide content = 79%). Aleurone is a single cell layer of wheat located between the starchy endosperm and the outer bran layers. The aleurone cells were separated from the pericarp layer and isolated to obtain a standard aleurone fraction. Cellulose (methyl-cellulose, Sigma–Aldrich) was used as a negative control substrate. All substrates were added to the fermentation medium at a concentration of 1% (wt/vol).

### Fecal Fermentation Experiments

Anaerobic (N_2_-sparged) batch fermentations were performed in triplicate using 10% fecal slurry (1% fecal inoculum) under controlled conditions [water-jacket vessels (Soham Scientific, Soham, United Kingdom), pH 6.8, temperature 37°C]. Basal medium contained per liter: 2 g peptone (Oxoid, Basingstoke, United Kingdom), 2 g yeast extract (Oxoid), 0.1 g NaCl (Fisher Scientific, Fair Lawn, NJ, United States), 0.04 g K_2_HPO_4_ (BDH, Toronto, ON, Canada), 0.04 g KH_2_PO_4_ (BDH), 0.01 g MgSO_4_7H_2_O (BDH), 0.01 g CaCl_2_6H_2_O (Honeywell, Morris Plains, NY, United States), 2 g NaHCO_3_ (Oxoid), 2 mL Tween 80 (Sigma–Aldrich, Oakville, ON, Canada), 0.05 g Hemin (Sigma–Aldrich) dissolved in 1 mL of 4 M NaOH (Fisher Scientific), 10 μL Vitamin K (Sigma–Aldrich), 0.5 g l-Cysteine HCL (Sigma–Aldrich), 0.5 g Bile Salts (Oxoid), and 4 mL of Resazurin (Sigma–Aldrich) (0.025 g/100 mL). Vessels were dosed with the substrates (1% wt/vol) after simulated *in vitro* upper gastrointestinal digestion and dialysis (Mandalari et al., 2008) and inoculated with 10% fecal slurry. The final volume of each culture was 200 mL. Samples were harvested at time points 0 (immediately after incubation) and 24 h.

### Fecal Microbiota Phylogenetic Profiling

Samples taken at T0 and originating from a same donor were pooled for further analysis. Briefly, DNA was extracted from 1 mL aliquots of fermentation effluent using the Fast DNA spin kit for feces (MP Biomedicals, Santa Ana, CA, United States). Fecal microbiota profiling was performed as described previously (Falony et al., 2016). The V4 region of the 16S rRNA gene was amplified with primer pair 515F/ 806R (GTGYCAGCMGCCGCGGTAA/GGACTACNVGGGTWTCTAAT, respectively) modified to contain a barcode sequence between each primer and the Illumina adaptor sequences to produce dual-barcoded libraries (Tito et al., 2017). Sequencing was performed on the Illumina MiSeq platform (MiSeq Reagent Kit v2, 500- cycles, 20% PhiX; Illumina, San Diego, CA, United States) according to the manufacturer’s specifications to generate paired-end reads of 250 bases in length in each direction. After de-multiplexing, fastq sequences were merged using FLASH (Magoč and Salzberg, 2011) software with default parameters, except for –min-overlap and –max-overlap which were set to 140 and 230, respectively. Successfully combined reads were filtered based on quality using seqtk trimfq with default parameters^1^. Chimeras were removed with the uchime2_ref algorithm of USEARCH (version 9.2.64) (Edgar et al., 2011). The taxonomy of reads was assigned using RDP classifier 2.12 (Wang et al., 2007) to generate phylum to genus level composition matrices. Bootstrap values from the RDP classifier were used to identify sequences with high-confidence genus assignments (bootstrap value > 0.8), while sequences classified with lower confidence were binned to the family assignment (labeled unclassified_family). To compare the different samples, sample counts were rarefied to 20,000 reads by random selection of reads and trimmed for the consequently absent OTUs with the *phyloseq* package (McMurdie and Holmes, 2013) in R version 3.3.0. In total, 18 samples, retrieved after 24 h of incubation, were analyzed covering 161 genera with an average of 65 genera per sample.

### Fluorescence *in Situ* Hybridization (FISH)

Genus-specific 16S rRNA targeted oligonucleotide probes labeled with the fluorescent dye Cy3 were used for enumerating bacteria. Fecal batch culture samples (375 μL) were fixed using cold 4% paraformaldehyde (Sigma–Aldrich) (pH 7.2) at a ratio of 1:3 (vol/vol) in a 1.5 mL Eppendorf tube and stored at 4°C between 4 and 16 h. Samples were centrifuged at 13,000 *g* for 5 min and washed twice (resuspending the pellet in 1 mL filtered PBS and subsequent centrifuging). The washed pellet was resuspended in a filtered-sterilized PBS/ethanol mix (1:1 vol/vol) and stored at -20°C for up to 3 months. The enumeration of microbial populations was carried out as described previously (Daims et al., 2005) using FISH-technique. Oligonucleotide probes used were Bif164 [specific for the *Bifidobacterium* genus (Langendijk et al., 1995)]; Bac303 [*Bacteroides* and *Prevotella* (Manz et al., 1996)], Chis150 [*Clostridium histolyticum* subgroup (Franks et al., 1998)], Erec482 [*Ruminococcus*–*Eubacterium*–*Clostridium* (EREC) cluster (Franks et al., 1998)], and Fpra655 [*Faecalibacterium* (Hold et al., 2003)]. Oligonucleotide EUB388 mix (Amann et al., 1990) was used for total bacteria enumeration, using 4′-6-diamidine-2-phenylindole (DAPI) staining as a control. Slides were enumerated using an Olympus microscope (Olympus, Shinjuku-ku, Tokyo, Japan) fitted with an EPI-fluorescence attachment and 15 randomized views (0,025 mm^2^, 100×) were counted for each sample.

### Gas Chromatography

Short-chain fatty acids (acetate, propionate, and butyrate) and branched short-chain fatty acids (isobutyrate, valerate, and methylbutyrate) were analyzed as described by Fava et al. (2012) with slight modifications to the method. Samples were acidified to pH 2–3 with 6 M HCl, centrifuged at 13,000 × *g* for 5 min, and filtered through a 0.2 mm polycarbonate syringe filter. Standard solutions containing 20, 10, 5, 1, and 0.5 mM external standards and 2 mM of internal standard (2-ethylbutyric acid) were used. Fatty acids were determined by gas chromatography on a Hewlett Packard (Agilent) 5890 Series II GC system (HP, Crawley, United Kingdom), fitted with a FFAP column (30 m × 0.53 mm, diameter 0.50 mm, J&W Scientific, Agilent Technologies, Ltd., South Queensferry, United Kingdom), and a flame-ionization detector. Glass wool was inserted in the injection port. The injected sample volume was 1 mL. Helium was used as carrier gas. The head pressure was set at 10 psi and the split ratio was 10:1. The flow rate of total gas was 140 mL/min. Injector and detector temperature were set at 280 and 300°C, respectively. The initial oven temperature was 100°C, maintained for 0.5 min, raised to 150°C at 81°C per min, then increased to 250°C at 50°C per min, and finally held at 250°C for 2 min. Fatty acid concentrations were calculated by peak integration using Atlas Lab managing software (Thermo Lab Systems, Mainz, Germany) and expressed as mM.

### Lactate Analyses Using Enzymatic Assays

Lactate was measured using the Lactate Assay Kit (Sigma–Aldrich) according to manufacturer’s instructions. Briefly, samples were centrifuged at 13,000 × *g* for 10 min and the supernatants were stored at -80°C, thawed on ice and filtered through 10 kDa MW cut-off columns (Millipore Amicon Ultra, Merck, Darmstadt, Germany). Two standard curves were used for each sample reading. Results are reported in mM.

### Gas Measurements

Gas production was measured through five replicate measures in separately conducted batch cultures (utilizing fecal samples from the same donors) and under anaerobic conditions using airtight serum bottles. Growth medium (pH controlled) [according to Rycroft et al. (2001)] was inoculated with freshly (anaerobic) prepared feces (1% wt/vol) and incubated anaerobically at 37°C for 24 h. Gas volume and pressure were measured at 3, 6, 9, 12, and 24 h using a transducer (Gems Sensors, Basingstoke, United Kingdom) according to the manufacturer’s instructions (Sarbini et al., 2011).

### Microbiome Analysis

Statistical analyses were performed in R version 3.3.0 (R Core Team, 2013). Observed richness was calculated with the R package *phyloseq* (McMurdie and Holmes, 2013). Microbiome variation between samples was determined by principal coordinates analysis (PCoA) using Bray–Curtis dissimilarity on the genus-level relative abundance matrix with the R package *vegan* (Oksanen et al., 2015) and visualized using the R package *ggplot2* (Wickham, 2009). Clustering analysis was performed based on hierarchical clustering with mean linkage using the R package *stats* (R Core Team, 2013). To assess microbiome variation between bran fraction and cellulose incubations, and between donors, (un)paired *t*-tests with Welch’s correction for unequal variances were carried out on log(1+×) transformed relative genus abundance data. Analyses were performed on core taxa, identified as annotated genera, present in at least 80% of samples, with abundance > 5% in at least one sample. The corresponding correlation effect sizes were calculated in R using the *lsr* (Navarro, 2015) and *compute.es* (Del Re, 2013) packages. Correction for multiple testing [Benjamini–Hochberg method (Benjamini and Hochberg, 1995), FDR] was performed. For the microbiome variation between bran fraction and cellulose incubations, FDR was applied for each substrate separately. Differences in observed richness between the different substrate incubation regimes (five-level categorical data) were assessed using non-parametric ANOVA (Kruskal–Wallis test using R package *stats*) and *post hoc* Dunn’s test (using R package *FSA*) for all pairs of comparisons between groups, with Benjamini–Hochberg adjustment for multiple testing (FDR).

### Donor/Substrate Effect Sizes in Microbiome Variation

Variation partitioning by stepwise distance-based redundancy analysis (dbRDA) was performed to determine how much of the microbial community profiles variation (Bray–Curtis dissimilarity) could be explained by the cumulative and individual contributions of substrate and donor, with significance calculated with a permutation test.

### Analysis of Metabolite Production

A Mantel test, based on Spearman’s rank correlation rho, was performed to test whether microbiome Bray–Curtis and metabolome Euclidean distance-based between-sample dissimilarities were correlated (1,000,000 permutations, R packages *vegan* and *ecodist*). Metadata was fitted on the PCA ordination (Supplementary Figure S5) using the prcomp function in the R package *stats*.

Normality of quantitative FISH and metabolite concentrations was assessed using the *car* package in R. As these metadata were normally distributed, no data transformation was applied. Differences between bran fraction and cellulose incubations were calculated using paired *t*-tests with Welch’s correction for FISH data and metabolite concentrations as described before. Correlations between relative genus abundances and metabolite data were analyzed using non-parametric Spearman tests for which correction for multiple testing [Benjamini–Hochberg method (Benjamini and Hochberg, 1995), FDR] was applied. The circos plot was constructed using the R package *circlize*.

## Conclusion

Here, we assessed the prebiotic potential of selected wheat bran fractions by performing a series of fecal slurry fermentations. We confirmed the bifidogenic effect of wheat bran fractions. The increase in *Bifidobacterium* spp. following ultrafine and soluble bran incubations was paired with a decrease in *Bilophila* relative abundances, matching *in vivo* observations on the prebiotic effect of inulin. In contrast with the more generalized effects observed upon pericarp bran fraction supplementation, aleurone fermentations selectively stimulated growth of butyrate-producing *Roseburia*, opening perspectives for its future development as a complementary prebiotic.

## Authors Note

The 16S rRNA gene amplicon sequencing results are available upon reasonable request.

## Author Contributions

LC, FF, GF, JV, and KT performed the conception and design of the study. LC and FF performed the data collection and experimental work. KD and SV-S performed the data preparation. KD, SV-S, and GF performed the statistical analysis, data analysis, and interpretation. KD, SV-S, GF, and JR drafted the manuscript. All authors performed the critical revision of the article and approved the final version for publication.

## Conflict of Interest Statement

Study products were provided by Cargill and JV is a Cargill employee. Cargill funded fermentation experiments and metabolite/microbiota analyses at FEM and sequencing/statistical analyses at VIB.

## Footnotes

1 https://github.com/lh3/seqtk
