# Supplementary material for: Prebiotic Wheat Bran Fractions Induce Specific Microbiota Changes
Source: Front Microbiol. 2018 Jan 24;9:31. doi: 10.3389/fmicb.2018.00031 (PMC5787670; doi:10.3389/fmicb.2018.00031)
Supplement: Supplementary file 2 [file Data_Sheet_2.docx]

Supplementary Material

**Prebiotic wheat bran fractions induce specific microbiota changes**

**Kevin D’hoe^†^, Lorenza Conterno^†^, Francesca Fava, Gwen Falony, Sara Vieira-Silva, Joan Vermeiren, Kieran Tuohy^†^, and Jeroen Raes^†,§^**

**^†^**These authors have contributed equally to this work.

**^§^ Correspondence:** Jeroen Raes: jeroen.raes@kuleuven.be

# Supplementary Data

# Supplementary Figures and Tables

Supplementary tables can be found in the Excel file entitled Data Sheet 1.XSLX

## Supplementary Figures


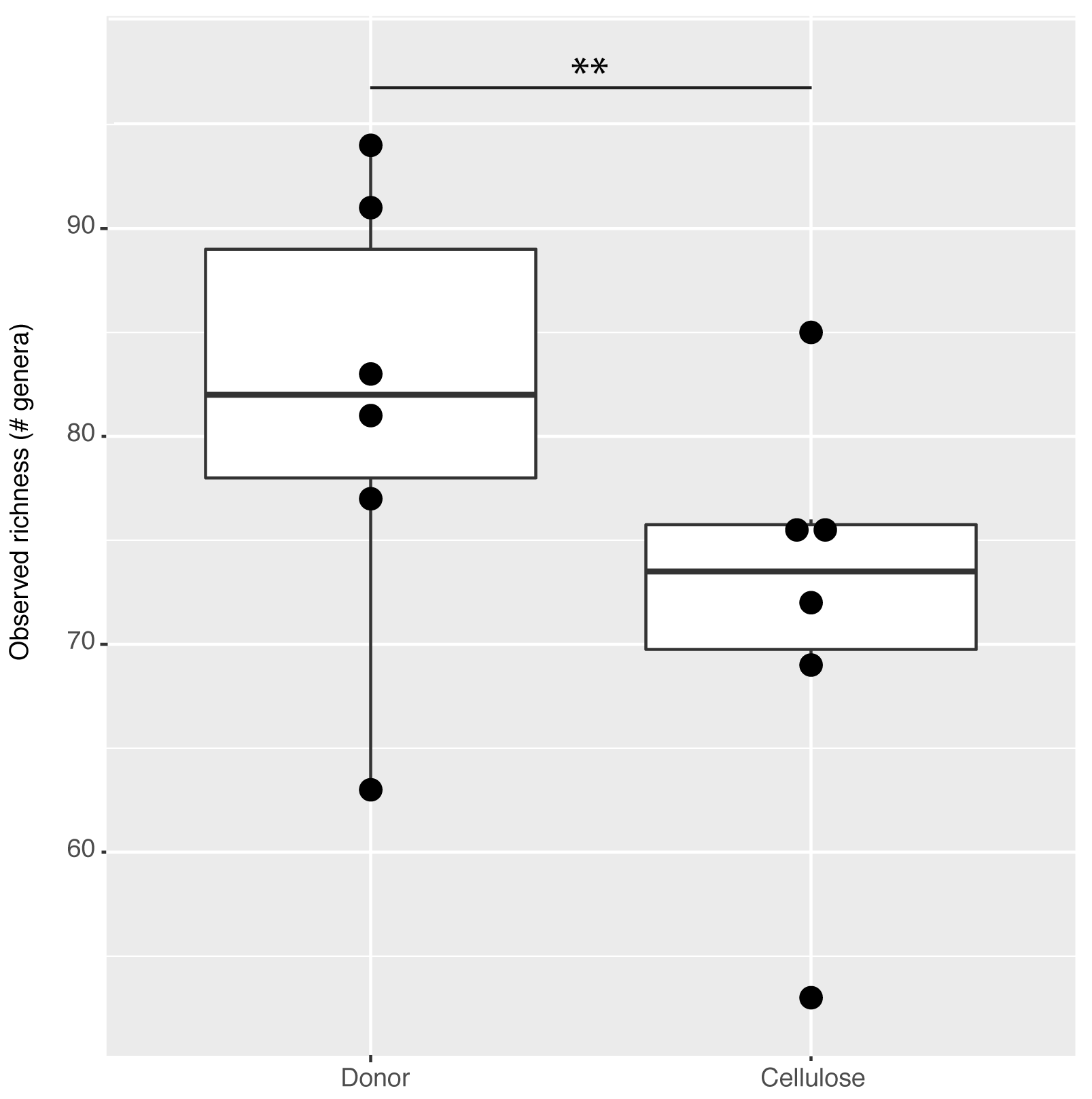


**Supplementary Figure S1.** Twenty-four hours cellulose fermentation resulted in a decrease of genus richness when compared to the initial donor material (paired t-test, effect size [ES] = -0.66, p-value = 8.1 10^-3^). The body of the box plot represents the first and third quartiles of the distribution, and the median line. The whiskers extend from the quartiles to the last data point within 1.5× IQR, with outliers beyond. [**] paired t-test, p-value < 0.01


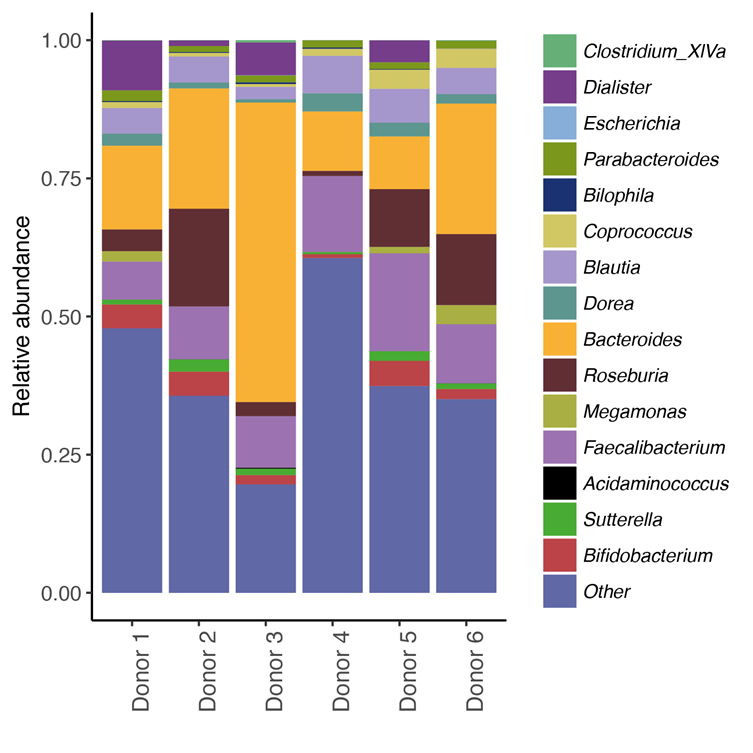


**Supplementary Figure S2.** Donor genus level relative microbiome composition of fecal slurries at the start of the fermentation experiments. The top 15 classified genera observed after 24 hours incubation (Figure 2) are depicted, with all others pooled into ‘Other’.


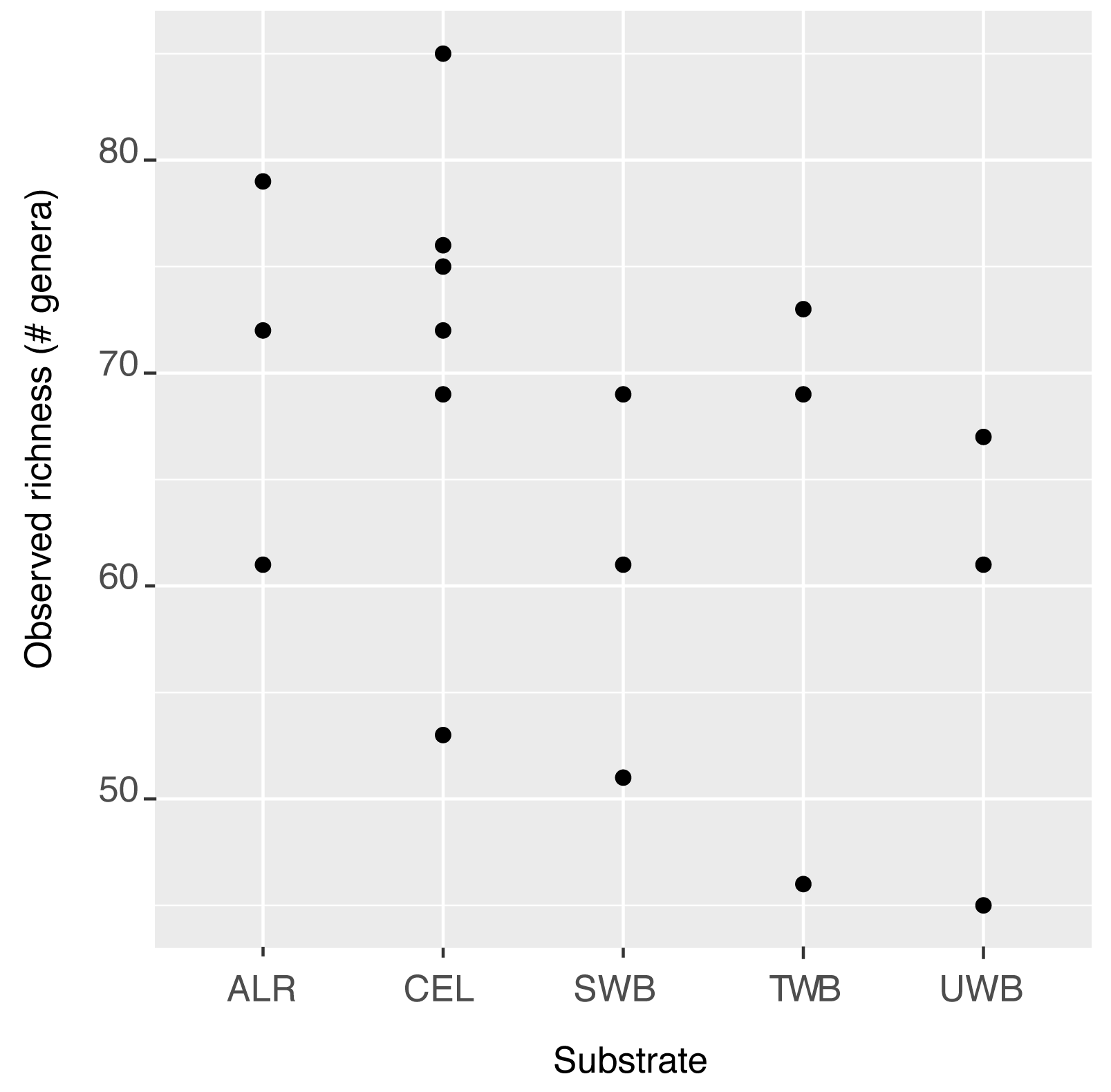


**Supplementary Figure S3.** After 24 hours of incubation with the substrates, no significant substrate-specific differences in observed richness were found (Kruskal-Wallis test, p-value = 0.21).


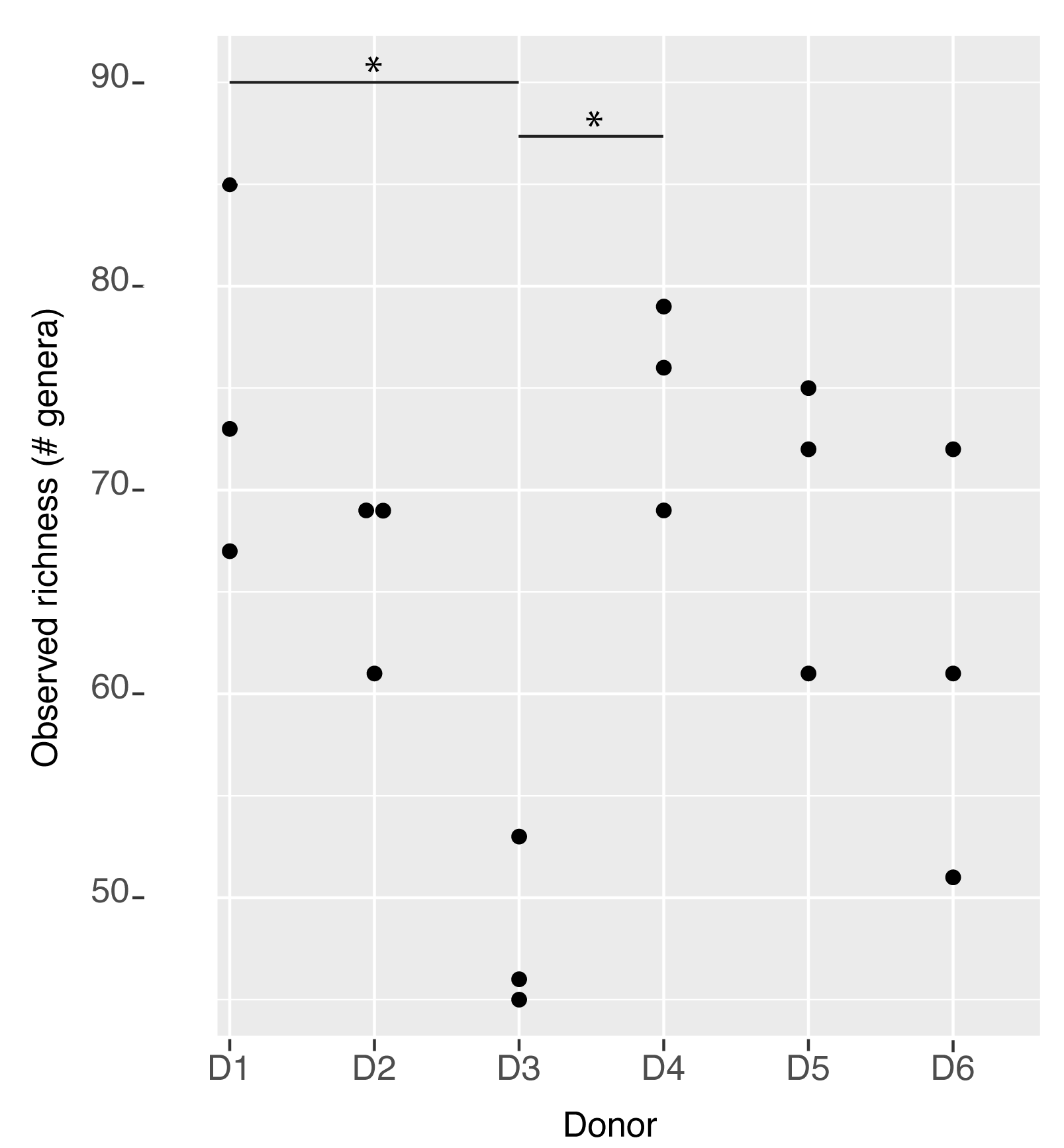


**Supplementary Figure S4.** Donor-specific differences in observed richness were still present after 24 hours of incubation with the substrates (Kruskal-Wallis test, p-value = 6.3 10^-2^; each data point represent richness observed after a single 24 hours bran fraction incubation). These were attributed to reduced richness in D3 incubations (Dunn’s test; Supplementary Table S5). [*] Dunn’s adj p-value < 0.1.


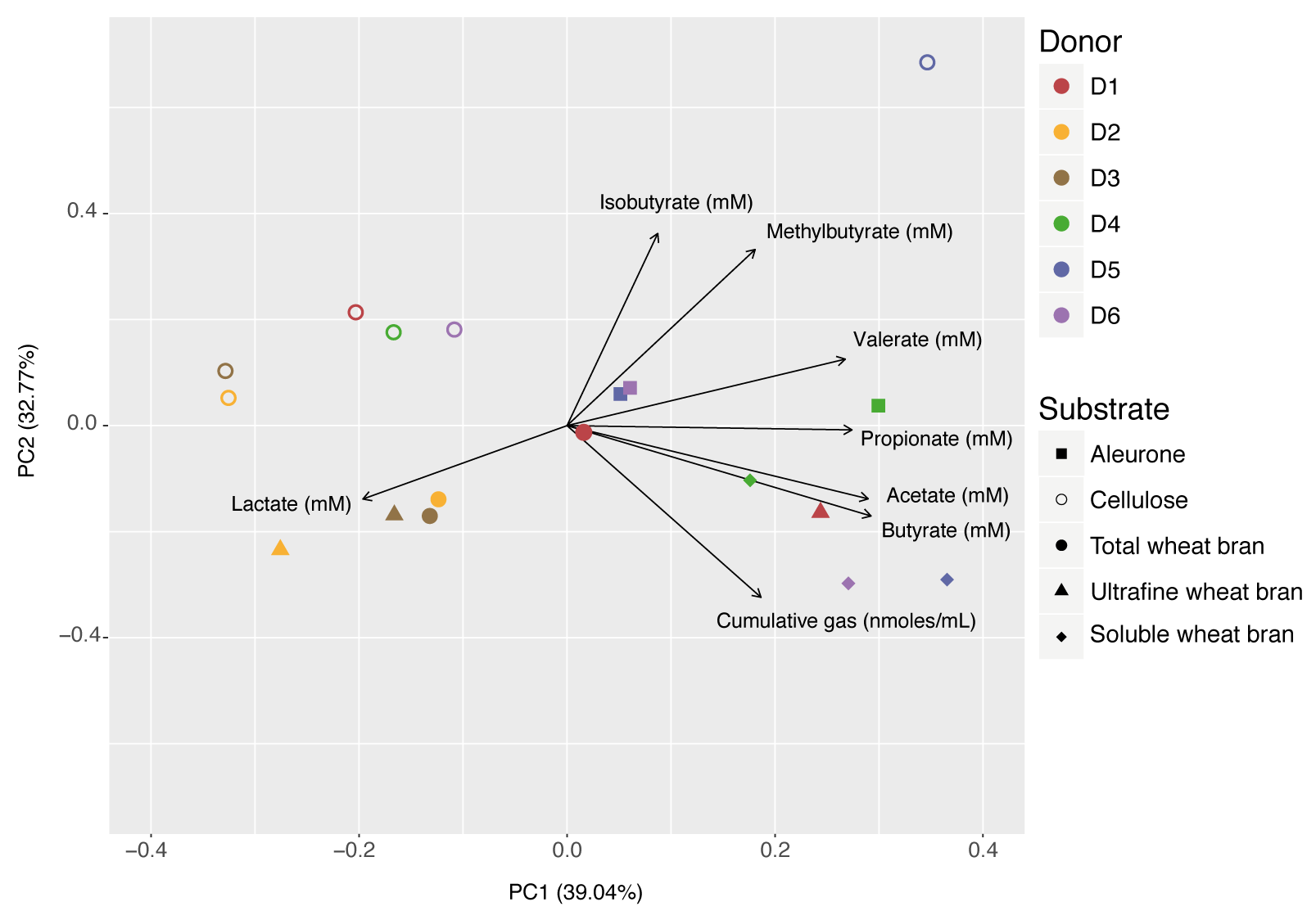


**Supplementary Figure S5.** Principal component analysis of metabolite profiles between fecal slurry incubation experiments after 24 hours. Points represent samples. Samples (n = 18) were colored and shaped by donor and substrate respectively. Blue vectors represent the metabolite variables (n = 8) used to build the PCs: the length of a vector reflects the contribution o­­f its associated variable in building the ordination space. The percentage of variance explained by the two first PC dimensions are reported on the axes.
